# Post-hepatectomy liver failure after major hepatic surgery: not only size matters

**DOI:** 10.1007/s00330-018-5487-y

**Published:** 2018-05-16

**Authors:** Ulrika Asenbaum, Klaus Kaczirek, Ahmed Ba-Ssalamah, Helmut Ringl, Christoph Schwarz, Fredrik Waneck, Fabian Fitschek, Christian Loewe, Richard Nolz

**Affiliations:** 1 Department of Bio-medical Imaging and Image-guided Therapy, Medical University of Vienna - Vienna General Hospital, Waehringer Guertel 18-20, A-1090 Vienna, Austria; 2 Department of Surgery, Medical University of Vienna - Vienna General Hospital, Waehringer Guertel 18-20, A-1090 Vienna, Austria

**Keywords:** Magnetic resonance imaging, Gadolinium ethoxybenzyl DTPA, Hepatectomy, Liver function tests, Liver failure

## Abstract

**Objectives:**

To compare the value of functional future liver remnant (functFLR) to established clinical and imaging variables in prediction of post-hepatectomy liver failure (PHLF) after major liver resection.

**Methods:**

This retrospective, cross-sectional study included 62 patients, who underwent gadoxetic acid enhanced MRI and MDCT within 10 weeks prior to resection of ≥ 4 liver segments. Future liver remnant (FLR) was measured in MDCT using semi-automatic software. Relative liver enhancement for each FLR segment was calculated as the ratio of signal intensity of parenchyma before and 20 min after i.v. administration of gadoxetic acid and given as mean (remnantRLE). Established variables included indocyanine green clearance, FLR, proportion of FLR, weight-adapted FLR and remnantRLE. functFLR was calculated as FLR multiplied by remnantRLE and divided by patient’s weight. The association of measured variables and PHLF was tested with univariate and multivariate logistic regression analysis and receiver operator characteristics (ROC) curves compared with the DeLong method.

**Results:**

Sixteen patients (25.8%) experienced PHLF. Univariate logistic regression identified FLR (*p* = 0.015), proportion of FLR (*p* = 0.004), weight-adapted FLR (*p* = 0.003), remnantRLE (*p* = 0.002) and functFLR (*p* = 0.002) to be significantly related to the probability of PHLF. In multivariate logistic regression analysis, a decreased functFLR was independently associated with the probability of PHLF (0.561; *p* = 0.002). Comparing ROC curves, functFLR showed a significantly higher area under the curve (0.904; *p* < 0.001) than established variables.

**Conclusions:**

functFLR seems to be superior to established variables in prediction of PHLF after major liver resection.

**Key Points:**

*• functFLR is a parameter combining volumetric and functional imaging information, derived from MDCT and gadoxetic acid enhanced MRI.*

*• In comparison to other established methods, functFLR is superior in prediction of post-hepatectomy liver failure.*

*• functFLR could help to improve patient selection prior major hepatic surgery.*

## Introduction

Liver resection is now an established method to prolong patient survival and which possibly results in a curative treatment option in selected patients with primary and metastatic liver tumours [1, 2]. The possibility of liver resection is determined by the technical feasibility of radical surgery and the capacity of the future liver remnant (FLR) to functionally compensate for tissue loss. The assessment of a patient’s eligibility for liver resection is, therefore, a delicate trade-off between oncologic radicalism and functional outcome. While an overly conservative approach might exclude individuals from curative surgery, a more aggressive strategy might put others at risk of post-hepatectomy liver failure (PHLF), which remains the major cause of perioperative morbidity and mortality [3]. Recent clinical guidelines base the indication for surgery on volume analysis and recommend that the FLR should be at least one-third of the total liver volume and 40–50% in patients with parenchymal liver disease [4, 5]. However, the relationship between liver volume and functional capacity is unpredictable and substantiates the inclusion of functional tests into the preoperative work-up. Serum markers and indocyanine green (ICG) clearance [5] cannot capture loco-regional differences in liver function, which might be crucial in the pre-hepatectomy setting. Scintigraphy-based tests (e.g. with ^99m^Tc-mebrofenin) expose patients to ionising radiation and are not widely available [5]. From a functional perspective, liver disease negatively affects the hepatobiliary uptake of gadoxetic acid, and the degree of relative parenchymal enhancement (RLE) may be used as an imaging biomarker of liver functionality [6–9]. Previous exploratory work suggested this marker as a potential predictor of PHLF [10–14].

On the basis of these observations, we hypothesised that the combination of FLR volume and RLE on gadoxetic acid enhanced MRI (i.e. functional future liver remnant, functFLR), adapted to a patient’s weight, might provide a more specific estimation of the FLR’s functional reserve. The aim of the present study was to benchmark this imaging biomarker against established clinical and imaging variables for the prediction of PHLF in a population that was undergoing major liver resection.

## Materials and methods

### Patient population

This was a single-centre, retrospective, cross-sectional study that was approved by the local institutional review board (No.1136/2017), which waived the need to obtain written, informed consent for data analysis. All patients who underwent pretreatment with gadoxetic acid (Primovist®/Eovist®, Bayer Health Care) enhanced 3-Tesla MRI and MDCT within 10 weeks prior to major liver surgery (i.e. at least four Couinaud liver segments), between January 2007 and December 2016, were eligible for analysis.

**Fig. 1:**
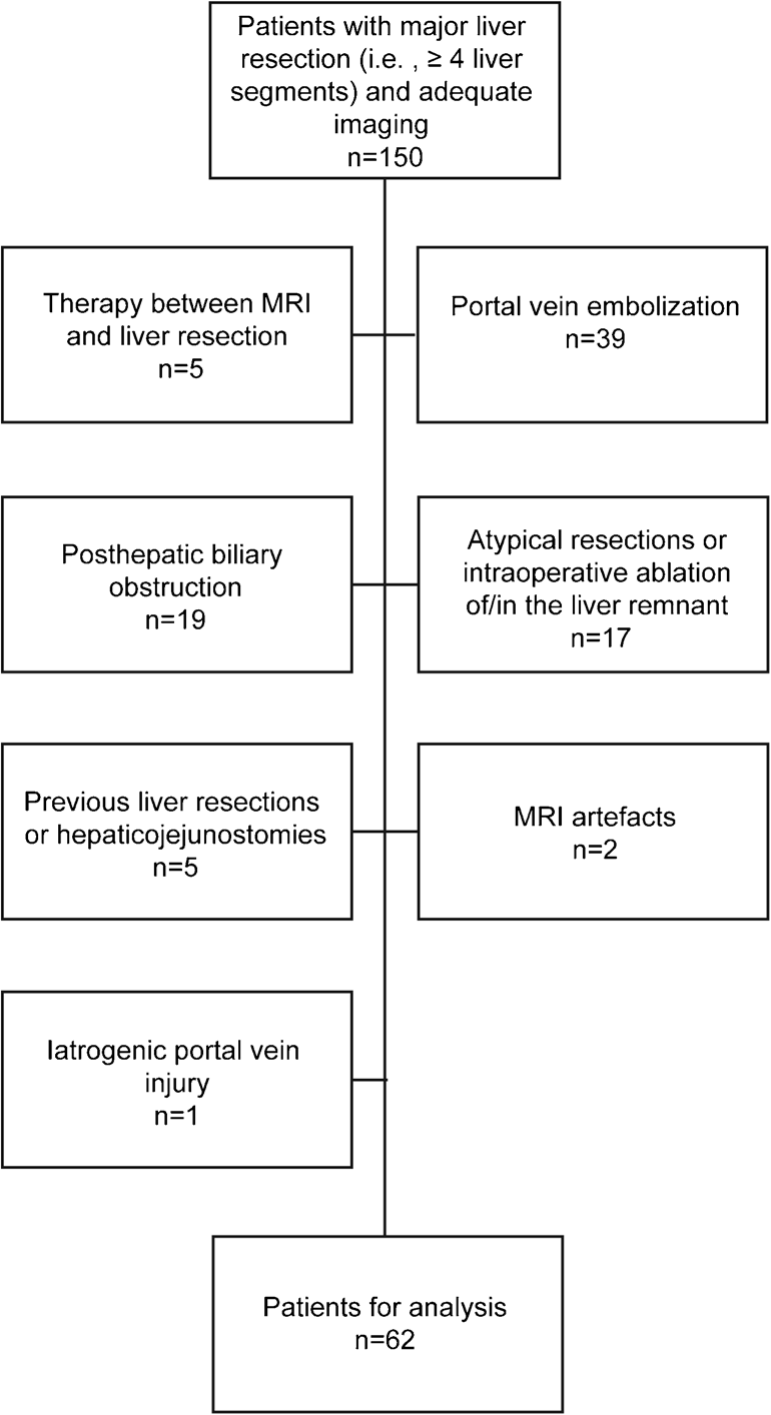
Flow diagram shows patient selection

Exclusion criteria were (1) treatment between imaging and liver resection (i.e. chemotherapy, transarterial chemoembolisation (*n =* 5) and portal vein embolisation (*n* = 39)); (2) signs of post-hepatic biliary obstruction (*n* = 19); (3) atypical resections or intraoperative ablation in the FLR (*n* = 17); (4) previous liver resections or hepaticojejunostomies (*n* = 5) and (5) MR imaging artefacts (*n* = 2). In addition, we had to exclude one patient who suffered from an iatrogenic portal vein injury with subsequent occlusion and liver failure (Fig. 1). Of the included patients, 22 subjects were part of a previously described cohort [10]*.* Compared to our study, analysis referred on the mean RLE of the whole liver without consideration of any volumetric data.

Age, sex, height, weight and body mass index were noted. In addition, plasma levels of bilirubin, creatinine, albumin, total protein, alkaline phosphatase, aspartate aminotransferase, alanine aminotransferase, gamma-glutamyltransferase, prothrombin time, haemoglobin, platelet count and leukocytes were noted preoperatively and at least until the 5th postoperative day or longer in case of PHFL.

### Multidetector CT protocols and volumetry

**Fig. 2:**
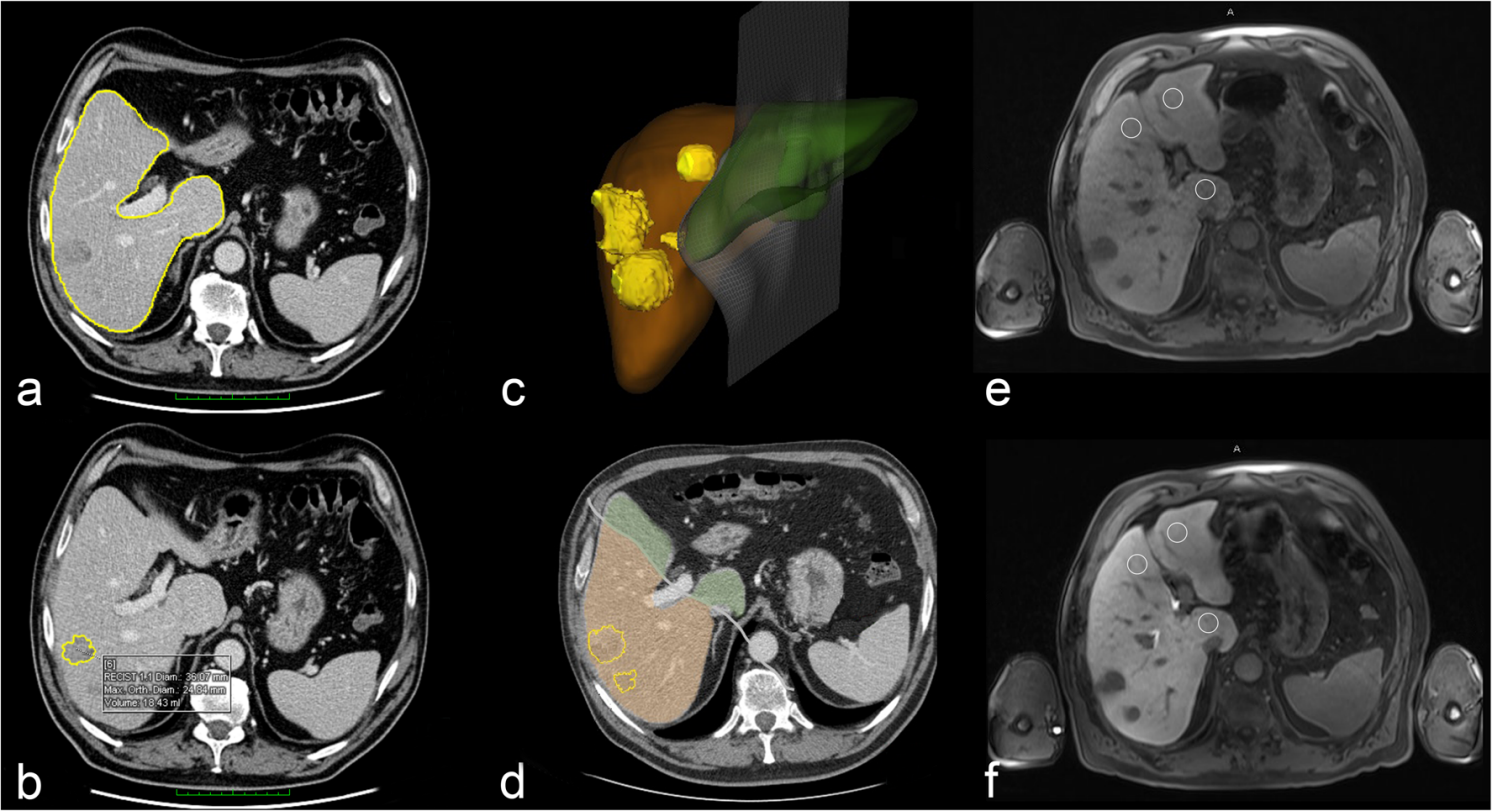
Imaging example of a 62-year-old male patient (weight 100 kg) with colorectal liver metastases in the right liver lobe, scheduled for a right hepatectomy. Volume analysis (**a**–**d**) exhibited a future liver remnant (FLR) of 757 ml. MRI revealed a mean relative enhancement of the future liver remnant (remnantRLE)—extracted from axial T1-weighted gradient echo MRI scans with fat suppression before (**e**) and 20 min (**f**) after intravenous injection of gadoxetic acid—of 0.66. As a result of the reduced uptake of the gadoxetic acid with reduced remnantRLE, the calculated functionalFLR was only 5 ml/kg. Postoperatively, the patient suffered from post-hepatectomy liver failure grade A, according to the grading system of the International Study Group of Liver Surgery (ISGLS)

All MDCT examinations were performed within our hospital. The minimum requirements for the imaging protocol included the following: (1) MDCT scanner with a patient size-adapted tube voltage (80–120 kVp); (2) active tube current modulation and (3) i.v. injection of 70–120 ml (depending on the body weight) of iodinated contrast agents (300–400 mg/ml iodine concentration) at a flow rate of 4–5 ml/s, followed by a saline flush of 20 ml using a power injector. Liver volumetry (Fig. 2a–d) was performed on transverse images using a soft-tissue kernel (B30F), section thickness and reconstruction interval was 3 mm/2 mm (*n* = 55) or 5 mm/4 mm (*n* = 7).

The syngo.CT liver analysis software (Siemens Healthineers) was used in a semi-automatic workflow for the following steps: loading and assignment of the unenhanced, arterial and venous phases (F.W., board-certified radiologist with 10 years of experience in abdominal imaging); automatic liver border segmentation with the option of manual correction was performed by the software for the venous phase only; automatic exclusion of the main portal vein, the hepatic venous confluence and the retrohepatic inferior vena cava from volume calculation. Focal liver lesions were marked by the reader, semi-automatically segmented and consecutively excluded from further volume analysis. The liver resection plane was determined on the basis of postoperative imaging studies or, if not available, on surgical reports of the particular patient (*n* = 13). All preoperative assessed liver volume measurements were blinded to the reader.

### MRI protocol and image analysis

MRI examinations were performed on a 3-Tesla unit (Magnetom Trio Tim®, Siemens Healthineers) equipped with a phased-array coil (placed ventrally on the upper abdomen) and a spine coil (for the dorsal part). Among others, the MR imaging protocol included unenhanced and enhanced fat-saturated T1-weighted sequences. These were conventional, three-dimensional, T1-weighted, spoiled gradient-echo volumetric interpolated breath-hold sequences with spectrally adiabatic inversion recovery fat saturation (VIBE SPAIR®, Siemens Healthineers) in the transversal plane (repetition time ms/echo time ms, 2.67/0.9; flip angle, 13°; bandwidth, 700 Hz/pixel; slice thickness, 1.5–2.0 mm, depending on patient size; average acquisition time, 19 s). Parallel imaging with an acceleration factor of 2 was used. The field of view was 350–400 × 350 mm for all transverse sequences, with individual adjustments depending on patient size. The enhanced T1-weighted sequences were performed after intravenous administration of 0.025 mmol/kg gadoxetic acid, using a power injector at a rate of 1.0 ml/s, and followed by a 20-ml saline flush with a bolus-tracking system. The hepatobiliary phase was performed 20 min after contrast injection [15].

MRI scans were retrospectively evaluated by a board-certified radiologist with 9 years of experience in abdominal imaging (R.N.), who was blinded to all clinical data. Images were reviewed on an IMPAX EE workstation (Agfa Healthcare). The signal intensity of the liver parenchyma was measured by manually placing a circular region of interest (area 2.0 cm^2^) in the middle of each Couinaud liver segment of the FLR, avoiding vessels, focal liver lesions and artefacts on the unenhanced and the hepatobiliary phase images (Fig. 2e, f). The RLE was calculated as the relative increase of liver parenchymal signal intensity according to the following formula:

$$ \mathrm{RLE}=\frac{\left(\mathrm{S}{\mathrm{I}}_{\mathrm{HB}}-\mathrm{S}{\mathrm{I}}_{\mathrm{unenhanced}}\right)}{\mathrm{S}{\mathrm{I}}_{\mathrm{unenhanced}}} $$

where SI_HB_ is the signal intensity in the hepatobiliary phase and SI_unenhanced_ is the signal intensity on the unenhanced scan. Subsequently, a mean RLE for all segments of the FLR (remnantRLE) was generated and used for further analysis.

### Variables used to predict PHLF

Established clinical parameters (plasma disappearance rate, PDR; median retention rate at 15 min, ICG-R15) were derived from indocyanine green (ICG) clearance testing, as described previously [16]. Imaging parameters provided by MDCT were FLR (ml), proportion of FLR (%) in relation to the whole liver volume and weight-adapted FLR (ml/kg). The remnantRLE, reflecting the mean RLE of the FLR, was derived from MRI. Finally, we created a combined imaging parameter defined as functional FLR (functFLR). The functFLR was calculated by the following formula:

$$ \mathrm{functFLR}=\frac{\mathrm{FLR}\ \mathrm{x}\ \mathrm{remnantRLE}}{{\mathrm{patient}}^{\prime}\mathrm{s}\ \mathrm{weight}} $$

### Liver surgery, PHLF, mortality and morbidity

Liver resection was performed or assisted by visceral surgeons with more than 10 years of experience in hepatobiliary surgery (K.K. and others), as described previously [17], using either a stapler or the Cavitron™ ultrasonic aspirator (CUSA™). PHLF was classified by the grading system of the International Study Group of Liver Surgery (ISGLS) [18]. Median hospital stay and cause of death within the hospital stay were noted. Postoperative morbidity was noted and rated according to the Clavien–Dindo classification [19].

### Statistics

Discrete variables were described with absolute and relative numbers and by using contingency tables; possible differences in discrete variables between groups were tested with the *x*^2^ test, and the Fisher exact test as appropriate. Continuous variables were described as medians and interquartile ranges (IQRs). Possible differences in continuous variables between groups were tested by the Wilcoxon test, the *t* test or the Kruskal–Wallis test, as appropriate. The association between established clinical and imaging variables and functFLR with outcome (presence of liver failure), defined as the reference variable, was tested by using logistic regression analysis with the calculation of odds ratios with 95% confidence intervals. Multivariate logistic regression analysis was conducted by using a stepwise selection of parameters, with a limit of *p* = 0.1 to enter and to stay in the model. The association of continuous variables was tested in a general linear model. In addition, the diagnostic ability of measured variables was described by using areas under the receiver operator characteristic (ROC) curves. The areas under the dependent ROC curves were compared according to the DeLong method [20]. No formal Bonferroni correction was applied in this exploratory study. *P* values are given as calculated and should be interpreted with care, considering alpha error accumulation. Cut-off values were defined with the Youden *J* statistics. Odds ratios were calculated to assess the association between the defined cut-off value and the presence of PHLF. Results were regarded as statistically significant if the probability of a type 1 error was less than 5% (*p* < 0.05). Statistical analyses were performed using SPSS for Windows (version 20.0; IBM Corporation) and MedCalc 12 (MedCalc Software).

## Results

### Study population

A total of 62 patients (31 female; 50%) with a median age of 59.8 (IQR 53.3–67.9) years were analysed. The median time from MRI and MDCT to liver resection was 0.4 (IQR 0.1–3.1) weeks and 3.6 (IQR 0.3–8.3) weeks, respectively. Detailed demographic and clinical data about the study population are given in Tables 1 and 2. Sixteen (25.8%) patients experienced PHLF according to the ISGLS criteria. Of these, nine (14.5%) patients did not need specific treatment, representing grade A liver failure, whereas six (9.7%) patients required changes in non-invasive clinical management (grade B). One patient subsequently needed invasive treatment and was classified as grade C liver failure, which accounts for 1.6% of all patients who undergo major liver resection.

**Table 1:** Patient characteristics separated for patients with and without PHLF (*n* = 62)

	Overall	No PHFL	PHFL	*p* value
Age, years	59.8 (53.3–67.9)	58.7 (50.1–69.0)	61.6 (56.7–67.0)	0.311
Height, m	1.72 (1.64–1.8)	1.69 (1.63–1.8)	1.76 (1.69–1.82)	0.200
Weight, kg	75.0 (63.8–85.0)	73.5 (62.0–84.0)	80.5 (73.8–96.3)	0.068
Body mass index, kg/m^2^	25.8 (22.5–28.0)	24.8 (22.3–27.8)	27.1 (24.4–29.1)	0.165
Quick test %	108.0 (93.5-128.5)	110.0 (91.5–129.0)	107.5 (94.8–127.5)	0.977
Haemoglobin, g/dL	13.6 (12.1–14.5)	13.4 (11.7–14.2)	14.2 (13.0–15.1)	0.022
Platelet count, G/L	238.0 (170.8–284.8)	236.0 (170.8–283.8)	249.5 (155.8–286.3)	0.872
Leukocytes G/L	7.0 (6.5–7.5)	7.0 (5.5–8.5)	6.7 (5.5–7.5)	0.294
Bilirubin, mg/dL	0.51 (0.41–0.80)	0.48 (0.37–0.73)	0.59 (0.46–0.92)	0.113
Creatinine, mg/dL	0.78 (0.70–0.92)	0.77 (0.68–0.88)	0.83 (0.7–0.98)	0.142
Albumin, g/L	43.3 (40.3–45.7)	43.3 (41.1–45.7)	43.2 (39.4–45.7)	0.489
Total proteins, g/l	74.3 (68.8–77.8)	74.9 (68.7–78.4)	73.5 (68.9–76.0)	0.404
Alkaline phosphatase, U/L	92.5 (69.5–118.5)	97.0 (68.0–123.0)	83.5 (71.8–114.3)	0.464
Aspartate aminotransferase, U/L	28.0 (24.0–35.3)	28.0 (23.8–35.3)	29.0 (24.5–39.0)	0.464
Alanine aminotransferase, U/L	22.0 (17.0–37.0)	21.5 (16.0–34.5)	24.0 (19.0–52.0)	0.280
Gamma-glutamyltransferase, U/L	55.0 (29.5–107.3)	56.0 (28.0–112.8)	50.5 (32.0–81.5)	0.766

Data are presented as median and interquartile ranges (IQR)

**Table 2:** Indications for major liver surgery (*n* = 62)

	Number	No PHFL	PHFL	*p* value
Malignant disease	51 (82.3%)	36 (58.1%)	15 (24.2%)	0.003
Colorectal liver metastasis	33 (53.2%)	24 (38.7%)	9 (14.5%)	0.009
Hepatocellular carcinoma	4 (6.5 %)	3 (4.8%)	1 (1.6%)	0.317
Intrahepatic cholangiocellular carcinoma	6 (9.7%)	5 (8.1%)	1 (1.6%)	0.102
Haemangioendothelioma	1 (1.6%)	1 (1.6%)	0	NC
Other liver metastasis	7 (11.2%)	3 (4.8%)	4 (6.4%)	0.705
Benign lesions	11 (17.7%)	10 (16.1%)	1 (1.6%)	0.007
Echinococcosis	6 (9.7%)	5 (8.1%)	1 (1.6%)	0.102
Liver adenoma	2 (3.2%)	2 (3.2%)	0	NC
Giant haemangioma	3 (4.8%)	3 (4.8%)	0	NC

*NC* not calculable

### Analysis of variables used to predict PHLF

The median values of variables that predict PHLF are given in Table 3. MDCT variables, remnantRLE and functFLR demonstrated significant differences between patients with and without PHLF. The comparison of established clinical variables (PDR and R15) in patients with and without PHLF did not show significant differences. These results were reflected in the univariate logistic regression analysis, where established MDCT variables, remnantRLE and functFLR were significantly related to the probability of PHLF (Table 4).

**Table 3:** Comparison of variables in patients with and without PHLF

	Overall, *n* = 62	No PHLF, *n* = 46	PHLF, *n* = 16	*p* value
Established clinical variables				
PDR, %	24.2 (18.0–27.7)	25.0 (18.0–30.3)	21.2 (17.8–24.7)	0.082
R15, %	4.0 (1.58–6.25)	2.75 (1.0–6.0)	4.25 (3.55–7.0)	0.075
Established MDCT variables				
FLR, ml	686.03 (497.67–931.02)	746.5 (563.7–986.0)	466.5 (403.4–736.0)	0.005
Proportion of FLR, %	41.5 (34.55–60.87)	49.3 (36.2–64.8)	33.9 (28.4–37.4)	< 0.001
Weight-adapted FLR, ml/kg	8.47 (6.73–12.89)	10.16 (7.45–13.93)	6.62 (5.31–8.0)	< 0.001
Established gadoxetic acid enhanced variable				
remnantRLE	1.21 (0.93–1.54)	1.35 (1.04–1.66)	0.91 (0.81–1.09)	0.001
Functional volume				
functFLR, ml/kg	10.92 (7.26–17.59)	12.93 (8.69–22.2)	6.44 (4.65–7.57)	< 0.001

Data are presented as median and interquartile ranges (IQR)

*PHLF* post-hepatectomy liver failure, *PDR* plasma disappearance rate, *R15* median retention rate at 15 min, *MDCT* multidetector CT, *FLR* future liver remnant, *remnantRLE* mean relative enhancement of the FLR, *functFLR* functional future liver remnant

**Table 4:** Results of univariate logistic regression analysis and receiver operating characteristic curves for the risk of liver failure according to the ISGLS criteria

	Odds ratio (95% CI)	*p* value	AUC (95% CI)	*p* value
Established clinical variable				
PDR	0.91 (0.82–1.01)	0.073	0.647 (0.51–0.783)	0.082
R15	1.13 (0.94–1.34)	0.190	0.350 (0.216–0.484)	0.075
Established MDCT variables				
FLR, ml	0.996 (0.994–0.999)	0.015*	0.736 (0.59–0.883)	0.005*
Proportion of FLR, %	0.906 (0.847–0.969)	0.004*	0.808 (0.692–0.924)	< 0.001*
Weight-adapted FLR, ml/kg	0.602 (0.430–0.843)	0.003*	0.825 (0.714–0.935)	< 0.001*
Established gadoxetic acid enhanced variable				
remnantRLE	0.029 (0.003–0.282)	0.002*	0.729 (0.672–0.912)	0.001*
Functional volume				
functFLR, ml/kg	0.994 (0.991–0.998)	0.002*	0.904 (0.803–0.977)	<0.001*

*CI* confidence interval, *AUC* area under the curve, *PDR* plasma disappearance rate, *R15* median retention rate at 15 min, *MDCT* multidetector CT, *FLR* future liver remnant, *remnantRLE* mean relative enhancement of the FLR, *functFLR* functional future liver remnant

*Significant difference

**Fig. 3:**
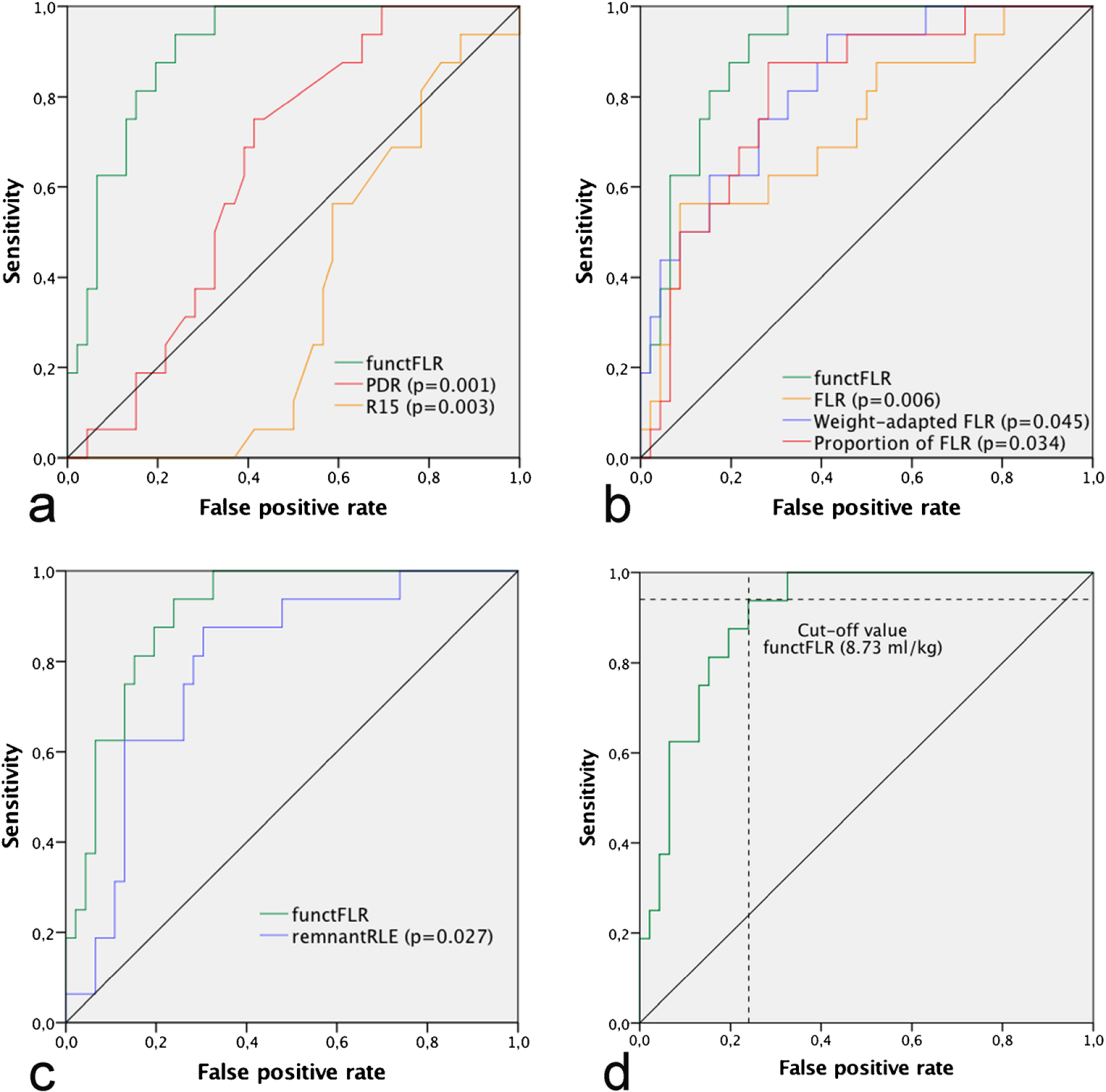
Receiver operating characteristic curves for the prediction of PHLF, comparing functFLR to **a** established clinical variables, **b** established MDCT variables and **c** an established gadoxetic acid enhanced variable. Corresponding *p* values are given in parentheses. **d** Optimal cut-off point defined as a functFLR of 8.73 ml/kg, demonstrated a sensitivity and specificity of 94% and 76%

Results of multivariate logistic regression analysis revealed that a decreased functFLR was independently associated with a higher probability of PHLF (0.561, 95% CI 0.389–0.807, *p* = 0.002). PDR, MDCT variables and remnantRLE also entered the model, but were rejected during stepwise selection. Comparing ROC curves, functFLR demonstrated a significantly higher area under the curve of 0.904 (95% CI 0.803–0.977; *p* < 0.001) than all other established variables (Fig. 3), followed by weight-adapted FLR, with an AUC of 0.825 (95% CI 0.714–0.935).

Median functFLR of patients without evidence of liver failure was 12.93 (IQR 8.69–22.2) ml/kg compared to 6.29 (IQR 5.20–7.50) ml/kg in patients with grade A liver failure and 6.34 (IQR 3.78–8.28) ml/kg in patients with grade B liver failure (*p* < 0.001). The only patient with grade C liver failure showed a preoperative functFLR of 6.59 ml/kg.

By means of functFLR and weight-adapted FLR, Youden’s *J* statistic revealed optimal cut-off points of 8.73 ml/kg and 9.49 ml/kg to separate patients with and without PHLF. PHLF was observed in 15 (57.7%) patients with a functFLR below the applied threshold and only one (2.8%) patient over the applied threshold, resulting in a sensitivity and specificity of 94% and 76%, respectively. The odds ratio for developing a PHLF was 47.7 (95% CI 5.7–403.5) higher in patients with a functFLR less than 8.73 ml/kg compared to a functFLR greater than 8.73 ml/kg.

### Postoperative in-hospital morbidity and mortality

During a median hospital stay of 11 (IQR 8–13.5) days, there were no deaths in our collective, resulting in an in-hospital mortality rate of 0%. Postoperative complications occurred in 24 (38.7%) patients. Using the Clavien–Dindo classification [19], we rated three (4.8%) patients as grade I, nine (14.5%) as grade II, six (9.7%) as grade IIIa and six (9.7%) as grade IIIb.

## Discussion

The present study demonstrated that the functFLR may provide a more accurate prediction of PHLF in comparison to ICG clearance, different established volumetric analyses, as well as the RLE in a population undergoing major hepatic resection. In multivariate analysis the functFLR was independently associated with a higher probability of PHLF and its area under the ROC curve was significantly higher than for the other established clinical and imaging parameters.

Currently, FLR volumetry is the method of choice for risk stratification in postoperative liver failure. Some authors have attempted to specify the demand of the FLR by calculating body surface area or body weight [21–25]. In this context, the FLR to body weight ratio was found to be superior to the ratio of FLR to liver volume in prediction of PHLF [25]. However, the quality of the parenchyma is not appraisable [26]. Although the most common preoperatively used quantitative liver function test in clinical practice [16, 27]—the indocyanine green clearance (ICG)—is able to reflect the global liver function [28, 29], it has been shown that there is no obligatory correlation with clinical outcome [30]. In our study, ICG clearance was not able to compete with other FLR estimation methods with regard to the prediction of PHLF.

Various studies have shown that gadoxetic acid uptake reflects liver function [8, 9, 31, 32]. The less the uptake, the more likely patients will suffer from PHLF [10, 13, 14]. Wibmer et al. demonstrated that a decreased RLE was independently associated with a higher probability of PHLF in patients undergoing liver resection of at least three segments [10]. Compared to our study, only the mean RLE of the whole liver was used for calculations, without consideration of any volumetric data. Several authors revealed that combining gadoxetic acid enhanced MRI parameters and volumetric data seems to be a promising tool in the preoperative work-up for liver surgery. Yoon and colleagues calculated a predicted liver remnant by multiplying the hepatic extraction fraction by the remnant volume, and correlated these results with post-treatment ICG-R15 [11]. They found a negative correlation, and concluded that gadoxetic acid enhanced MRI enables prediction of postoperative liver function. For the assessment of hepatic extraction fraction [33], dedicated software is mandatory, which made this method impracticable [34]. In addition, some authors used an indirect test for liver function (ICG-R15) as an outcome variable because none of their patients suffered from PHLF [11].

Itoh et al. assessed the functional liver remnant by MDCT volumetry—normalised to the body surface area—and the liver enhancement in gadoxetic acid enhanced MRI [12]. Compared to our study, instead of remnantRLE, the remnant function was calculated as the ratio of the liver to the psoas muscle on the hepatobiliary phase and on the pre-contrast phase. Additionally, in their collective more than 80% of patients underwent resection of less than two liver segments, and only three patients suffered from PHLF. In multivariate analysis, the calculated functional liver remnant volume was an independent predictor for liver-related morbidity, albeit not specifically for PHLF [12]. For patients undergoing major liver resection, the applicability of functFLR is supported by our results, where functFLR was the only independent predictor of PHLF in the multivariate analysis. In addition, the superiority of functFLR in terms of the prediction of PHLF was supported by a significantly higher AUC (0.904) compared to all other tested clinical and imaging parameters.

The favoured cut-off value for the FLR is given with a weight-adapted FLR (i.e. FLR to body weight ratio) of at most 0.5% (i.e. 5 ml/kg), with a sensitivity and specificity of 100% and 84.1% for the prediction of death from PHLF [24]. In our study, the cut-off value for weight-adapted FLR was higher (9.49 ml/kg), presumably because our definition of PHLF includes all grades of liver dysfunction. However, in our study the weight-adapted FLR volume revealed a significantly lower AUC compared to functFLR.

In contrast to the common definition of major hepatectomy (at least three Couinaud liver segments), we included only patients with resection of at least four liver segments, since these patients present a higher risk of PHLF. The incidence of PHLF, at about 26%, is higher than the described 0.7–9.1% in the literature [3], as most of our patients (nine of 16 patients) had a minor PHLF (grade A). The in-hospital mortality rate in our collective was 0%. However, we excluded one patient who died because of an intraoperative iatrogenic portal vein injury. Apart from that, postoperative complications occurred in about 39% of our included patients, which is consistent with the described 44% [35].

The major limitation of our study was its retrospective design. Second, compared to other studies [36], our collective consisted mostly of patients with colorectal liver metastases. Hence, we did not assess the Child–Pugh score and the model for end-stage liver disease score, which is reasonable because only one of our patients suffered from chronic liver disease. The reason for the low hepatocellular carcinoma (HCC) incidence in our collective may be because most patients with HCC have a limited functional liver reserve and, consequently, often need preoperative liver volume augmentation before major liver surgery. Thus, the results of our study cannot be applied to patients with liver cirrhosis and HCC, where preoperative assessment of liver function is especially important.

## Conclusion

A decreased functFLR is independently associated with a higher risk of liver failure after major liver resection. Compared to established preoperative methods, the functFLR seems to be more accurate in the prediction of PHLF. This combination of volumetric and functional information could help to optimise preoperative patient selection.

## Abbreviations

AUC: Area under the curve
CI: Confidence interval
functFLR: Functional future liver remnant
FLR: Future liver remnant
ICG: Indocyanine green
ICG-R15: Indocyanine green 15-min retention rate
MDCT: Multidetector computed tomography
MRI: Magnetic resonance imaging
PDR: Plasma-disappearance rate
PHLF: Post-hepatectomy liver failure
remnantRLE: Mean RLE of the FLR
RLE: Relative liver enhancement
ROC: Receiver operator characteristics
